# Antipsychotic-induced tardive dyskinesia management: A case report

**DOI:** 10.1192/j.eurpsy.2026.12131

**Published:** 2026-07-31

**Authors:** A. Souidi, S. Belbachir, A. Ouanass

**Affiliations:** Ar-razi hospital, Rabat, Morocco

## Abstract

**Introduction:**

Tardive dyskinesia (TD) is an iatrogenic disorder characterized by a range of movement abnormalities, described as irregular, stereotyped, and choreiform caused by exposure to antipsychotics. This condition can lead to significant or disabling symptoms that affect quality of life. However, the exact mechanism underlying TD remains unclear. Pharmacotherapy of TD includes cholinergic drugs, benzodiazepines, antioxidants, amantadine, propanolol, botulinum toxin, whereas the non-pharmacotherapy approach includes modified electroconvulsive therapy and deep brain stimulation.

We successfully treated a chronic schizophrenia patient with comorbid TD using Aripiprazole and botulinum toxin after trying degression, switch of antipsychotics and association with calcium channel blockers.

**Objectives:**

To determine the effects of calcium channel blocker drugs (Amlodipine) for treatment of neuroleptic-induced tardive dyskinesia in people with schizophrenia, and the efficiency of botulinum toxin in treating induced tardive dyskinesia.

**Methods:**

through a case report .

**Results:**

A 22 years male, diagnosed with schizophrenia since the age of 16 years, with one hospitalization. The evolution of his disease was marked by the development of a tardive dyskinesia, cervical and brachial movements disorders with rapid worsening in few months. During his follows-up, we tried switching the incriminated antipsychotics, adding benzodiazepines and vitamin E, before trying the calcium channel blocker for two months, to end with the botulinum toxin.

**Conclusions:**

Antipsychotic drugs are known to cause a variety of adverse effects, including tardive dyskinesia. Hence the importance of knowing the pharmacotherapy and non-pharmaco- therapy to manage this effect, through this case report where it tardive dyskinesia got treated with a spectacular improvement in abnormal involontary movements scale .

**Disclosure of Interest:**

None Declared

